# Mr. Woodham in Reply to Mr. Walker

**Published:** 1817-04

**Authors:** 


*For the London Medical and Physical Journal.*

### Mr. Woodham *in Reply to Mr. Walker.*

"Let the gall'd jade wince."

"ECCE iterum Crispinus!" *(Pedantry.)* Mr. Walker again upon the stage! I fancy, I hear your readers exclaim on viewing your last Number. His *fifth* accusation is, that I am a churl, and a man of an immature or distempered judgment, and a poor deluded gentleman, and, to complete the climax, troubled with the spleen.

When Rousseau was informed, that the Inquisition had ordered one of his books to be burnt, he replied, "bruler n'est pas repondre." (More *pedantry.*) Calling names, I conceive, is not answering. With regard to being splenetic, he may believe me, when I say, that I am

"Spleen, vapours, and *small-pox,* above them all."

Allow me, Mr. Editor, (I hope you have pardoned my *gross* reprobation) a few words, "Brevis esse laborabo." (More *pedantry* still.)

"When this gentleman," (meaning myself,) Mr. Walker observes, "fastens upon any one, methinks he is not easily choaked choaked off;"—To talk of a gentleman *fastening* upon any one, and that he is not easily *choaked* off, may be considered very *elegant* and *polished* phraseology at Oxford; in London, it would be deemed somewhat *vulgar*. Had Mr. W. said, "when once this gentleman begins to criticise, it is not an easy task to make him desist from his criticisms," he would have expressed himself, if not justly, at least not vulgarly. Fastening on, and choaking off, are terms in use here only among Exeter-Change men, buffers, and gentlemen of the *Fancy*. Mr. W. goes on, "I may in future, (that is, I do now,) venture to attribute Mr. Woodham's *churlish remarks* to an immature or distempered judgment." Whether my remarks on his theoretical and practical *improvements*, more particularly in small-pox, (a disease for which he seems to have a more than parental fondness,) were churlish, and the effects of the cause he has assigned, I shall leave the public to determine. Mr. W. continues, "Mr. Woodham (poor deluded gentleman!) seems entirely to have mistaken the design of my communications, and, as I apprehend, his own qualification, which is, that he should *profit* by them, not *comment* on them." How *immature*, or how *distempered* must be that *judgment*, and how *deluded* by an over-weening opinion of his own abilities must he be, who could imagine that myself, or even a mere novice, could profit from dull and prolix relations of absurd hypotheses and jejune observations! or was in want of that routine information, which was to be found, as I before mentioned, in every system and compendium of medical practice!

*London; March* 5, 1817.

---

**[Our only apology for inserting this monthly spar, or this contention *versibus alternis* is, that the communications are short, and that each party contrives some little novelty for the other to answer. We hope, however, both will see the propriety of hereafter submitting to the decision of the public, who must by this time be ample judges of the comparative merits of each, as men of judgment or men of wit.—Edit.]**

